# Ultrasound-Mediated Ocular Drug Delivery: From Physics and Instrumentation to Future Directions

**DOI:** 10.3390/mi14081575

**Published:** 2023-08-09

**Authors:** Blair Duncan, Raida Al-Kassas, Guangming Zhang, Dave Hughes, Yongqiang Qiu

**Affiliations:** 1 School of Engineering, Faculty of Engineering & Technology, Liverpool John Moores University, James Parsons Building, Byrom Street, Liverpool L3 3AF, UK; 2 School of Pharmacy & Biomolecular Sciences, Faculty of Science, Liverpool John Moores University, James Parsons Building, Byrom Street, Liverpool L3 3AF, UK; 3 Novosound Ltd., Biocity, BoNess Road, Newhouse, Glasgow ML1 5UH, UK

**Keywords:** ultrasound, targeted drug delivery, ocular drug delivery, ocular barriers, acoustic cavitation, acoustic streaming

## Abstract

Drug delivery to the anterior and posterior segments of the eye is impeded by anatomical and physiological barriers. Increasingly, the bioeffects produced by ultrasound are being proven effective for mitigating the impact of these barriers on ocular drug delivery, though there does not appear to be a consensus on the most appropriate system configuration and operating parameters for this application. In this review, the fundamental aspects of ultrasound physics most pertinent to drug delivery are presented; the primary phenomena responsible for increased drug delivery efficacy under ultrasound sonication are discussed; an overview of common ocular drug administration routes and the associated ocular barriers is also given before reviewing the current state of the art of ultrasound-mediated ocular drug delivery and its potential future directions.

## 1. Introduction

Through a combination of advancements in electronics, material science, and signal processing, modern ultrasound devices have developed from their roots in SONAR into a versatile technology that is fundamental to many scientific and industrial processes, as well as being integral to many everyday devices [1,2,3,4,5]. Today, ultrasound is used for imaging, flaw detection, surface cleaning, navigation, ranging, and haptics, among other applications across numerous fields [6,7,8,9,10,11]. However, it is perhaps most synonymous with its medical applications, particularly in medical imaging. Medical ultrasound imaging is a non-invasive means of obtaining and conveying information about organ morphology, pathology, and motion, with many advantages such as relatively low cost, real-time imaging, and not using any ionizing radiation [12,13,14]. Ultrasound can also be used for therapeutic purposes and has been used in fields such as physiotherapy, oncology, urology, and ophthalmology for many years [15,16,17,18]. More recently, the thermal and non-thermal bioeffects generated by ultrasound have been examined for the potential of enhancing drug delivery [19,20,21].

The inability to achieve therapeutically effective concentrations of drugs at the site of actions not only undermines the treatment effort but can also expose otherwise healthy tissues to cytotoxic effects. Therefore, a method of attaining a more confined spatial distribution of administered drugs has been highly sought after. The development of tailored drug carriers for active and passive targeting has been effective at localizing drugs to specific regions of the body that exhibit pathologically induced anatomical and physiological alterations [22,23]. However, there are still some limitations of this method that merit the further investigation of other technologies to control the distribution and action of drugs in vivo.

Several systems have been proposed and tested for instigating the release of drugs from carriers or transiently altering the structure of biological membranes to allow the passage of therapeutic agents. The deposition of energy in biological tissue by emission of radiation (acoustic or electromagnetic) or by the application of fields (electrical or magnetic) can bring about changes in the structure or function of either drug carriers administered within or on the tissue, or to the tissue and constituent cells [24,25,26]. The advantage of these systems is that the stimulation source is not local to the site of drug action, as is the case with carriers that are sensitive to pH or the action of biomolecules, but rather can be applied externally from outside the body for non-invasive drug delivery [27,28]. One such technology that has been increasingly scrutinised for this application is ultrasound. Through both thermal and mechanical effects, ultrasound is effective in increasing drug delivery efficacy in a variety of tissues and medical fields [29,30].

In this review, we discuss the bioeffects induced by ultrasound and how they have been exploited to increase drug delivery efficacy, with particular emphasis on ocular drug delivery. The fundamental aspects of ultrasound physics are detailed along with the thermal and non-thermal effects they incite in biological tissue. A brief overview of the instrumentation of diagnostic and therapeutic ultrasound is also given in this review. The ultrasound-induced bioeffects responsible for the enhancement of drug delivery and the organs on which this technique has been demonstrated are discussed. Furthermore, attention is given to the ocular barriers that inhibit drug delivery to various tissues of the eye and the existing delivery methods that are used to overcome these barriers. We also describe the currently available practices for ultrasound-mediated ocular drug delivery to the anterior and posterior segments of the eye. Finally, some of the challenges and limitations of ultrasound-mediated ocular drug delivery are discussed, and possible future directions of the method are proposed.

## 2. Ultrasound Physics and Bioeffects

### 2.1. Properties of Sound Waves

Ultrasound is a mechanical wave that travels through a compressible medium as a region of increased pressure generated by small oscillations of the constituent molecules [31]. The number of oscillations per unit of time is called the frequency of the wave, with the term “ultrasound” being designated to waves with frequencies of ≥20 kHz and therefore beyond the upper threshold of human hearing [32]. The inherent properties of sound waves that define their nature are the relative direction of displacement of the molecules, their speed in a given medium, frequency, pressure amplitude, and energy content. Other parameters may be derived from these quantities [33]. The first of these characteristics can be used to classify the wave as longitudinal (compressional), transverse (shear), or other types of waves. Longitudinal waves are distinguished by the fact that the local particle velocity is parallel to the direction of wave propagation, whereas transverse waves are characterised by the oscillation of the particles orthogonal to the direction of energy transfer.

The wave speed is dependent on the density (ρ) and stiffness (*k*) of the medium in which the wave travels as well as the temperature of the medium, a fact that is used for ultrasound thermometry [34]. It was shown by Sarvazyan et al. [35] that the longitudinal speed of sound in skeletal muscle increased non-linearly with increasing temperature. For a constant temperature however, wave speed (*c*) can be approximated as a function of the aforementioned physical properties of the medium by [33]

(1) $$c=\sqrt{\frac{k}{\rho }}.$$

It is noticeable from Equation (1) that in this approximation the wave speed is considered independent of the frequency and pressure amplitude of the wave.

The pressure amplitude of a sound wave is often taken as the pressure difference between the region of compression (or rarefaction) and the ambient pressure of the medium in its unperturbed state [33]. These fluctuations in pressure and the associated changes in the local density of the medium and particle velocity are responsible for the transfer of energy through the medium by the sound wave. The energy per unit volume (energy density) attributed to the sound wave is given by the expression [36,37,38,39]

(2) $$E_{\rho }=\frac{1}{2}\frac{p^{2}}{c^{2}\rho }+\frac{1}{2}\rho v^{2}.$$

Here, *p* is the acoustic pressure (excess pressure) and *v* is the local velocity. The acoustic energy flux density, or energy transmitted through a unit area in unit time, is called the acoustic intensity [37], and the product of the acoustic intensity and the area of a surface through which the wave travels gives the acoustic power, a critical parameter in therapeutic ultrasound. Since, in a sound wave, a particle of the medium will undergo oscillatory motion about its equilibrium position, its displacement may be expressed as *s* = *s*_0_sin(*ωt*−*kx*), with *s*_0_ being the maximum displacement magnitude, *ω* the angular frequency, *k* the wave number, and *x* the distance of the particle’s equilibrium position from the sound source [40]. The particle velocity is then the time derivative of this expression, *v* = *ωs*_0_cos(*ωt*−*kx*) [41]. From this, and Equation (2), it can be seen that the energy density and thus the intensity of the wave can be increased by increasing the acoustic pressure or the wave frequency. Additionally, the intensity of the wave may be increased by confining the energy to a smaller volume by focusing the wave. This can be achieved with lenses, electronic scanning of multiple elements arranged in an array, or with specially designed focused transducers [42,43,44].

### 2.2. Wave–Matter Interactions

The acoustic impedance of a material is the measure of the resistance of the material to the propagation of sound waves and is quantified by [33,45]

(3) $$z=\frac{p}{v}$$

where *p* is the local pressure at a point in the material body and *v* is the local velocity caused by that pressure force. Here, *z* is known as the specific acoustic impedance and has the dimensional formula of [kg·m^−2^·s^−1^]. This is the dimension of density multiplied by speed, and thus the product of a material’s density by its longitudinal speed of sound is termed the characteristic acoustic impedance (Z=ρc). When sound waves encounter boundaries between media with different acoustic impedances, such as between the layers of the eye (see Table 1), a portion of the energy is transmitted into the second medium whilst some of the energy is reflected into the first medium. The proportion of the energy reflected at the boundary is related to the acoustic impedances of the interfacing media by [46]

(4) $$R=\frac{Z_{2}-Z_{1}}{Z_{2}+Z_{1}}$$

and thus the proportion of energy transmitted into the second medium is

(5) T=1−R.

With knowledge of the speed of sound in a material, the temporal distribution of the reception of wave reflections can be used to obtain the spatial distribution of the interfaces causing those reflections. This is the process carried out in ultrasound imaging and flaw detection and is an extremely useful tool in numerous fields. Although, as the mechanical properties of biological tissue are known to be altered with disease progression, so too is the speed of sound and thus the acoustic impedance of the affected tissue. This can result in imaging artifacts and invalid distance measurements. As an example, ultrasound is commonly used in ophthalmology for measuring eye axial length [47]; however, myopia, glaucoma, age-related macular degeneration, and dry eye disease are all ocular conditions that have been found to result in changes to the mechanical properties of the eye and therefore the speed of sound [48,49]. Normal speed of sound values for various ocular tissues, as reported by Thijssen et al. [50], can be seen in Table 1. With refraction of sound waves also occurring when the wave impinges upon the interface of two media, the curvature of the eye can lead to refraction artifacts [51], errors in targeting the ultrasound beam for therapeutic applications, and potentially misdirection of acoustic radiation forces generated for targeted drug delivery.

**Table 1:** Acoustic properties of various ocular tissues [50].

Tissue	Cornea	Vitreous	Lens	Retina	Choroid	Sclera
Speed of sound (m·s^−1^)	1553 ± 3	1506 ± 3	1620 ± 3	1538 ± 20	1527	1583 ± 10
Acoustic Impedance (kg·m^−2^·s^−1^) 10^6^	1.59 ± 0.03	1.51 ± 0.01	1.71 ± 0.01	1.55 ± 0.09	1.53	1.66 ± 0.02

#### 2.2.1. Thermal Effects

In a real medium, the existence of internal friction (viscous) forces will result in the dissipation of the acoustic energy into internal energy or heat, which may induce thermal effects in biological tissues [41]. In the eye, tissues that may be particularly susceptible to ultrasound-induced temperature increases are the cornea, lens, and vitreous humour. This is due to both the high collagen content, which is an effective absorber of acoustic energy, in the former two tissues and also the lack of blood perfusion in these tissues, limiting the dissipation of heat [52].

Focusing ultrasound waves into a confined volume can generate localised hyperthermia with temperatures exceeding 65 °C [53]. Both mild and more extreme ultrasound-induced tissue heating have been used to reduce fracture healing time, relieve joint stiffness, ablate malignant tissues and, more recently, achieve targeted drug delivery in combination with temperature-sensitive drug carriers for spatially and temporally controlled release [54,55,56]. In particular, temperature-sensitive liposomes have been one of the most frequently studied drug carriers for this technique [57,58,59,60,61,62,63,64,65,66,67].

One parameter of an ultrasound system configuration that is frequently referred to when determining the safety of the procedure is the thermal index, which is defined in terms of acoustic power. It is the ratio of the output power of the ultrasound system to the power required to raise the temperature of the sonicated biological tissue by 1 °C [68]. Due to the adverse effects that excessive ultrasound power and intensity can have, the system must be calibrated correctly. This is usually achieved with acoustic radiation force balances or calorimetric methods to determine the power and intensity of the sound field [69,70].

#### 2.2.2. Mechanical Effects

The mechanical effects that ultrasound causes in biological tissues can result from acoustic radiation forces, fluid-streaming-induced stresses, or acoustic cavitation. The acoustic radiation force and acoustic streaming forces are generated by pressure gradients created by the sound wave, the advection of momentum by the motion of the fluid particles, and attenuation of the wave [71,72,73]. Ultrasonic cavitation, on the other hand, refers to cyclic compression and rarefaction of a fluid in an ultrasonic field, causing the formation and volumetric oscillation (or collapse) of cavities. Cavitation effects can be harnessed for therapeutic applications (see Section 3) but, if unregulated, can lead to large temperature and pressure gradients that can be destructive to biological tissues [74,75].

The likelihood of mechanical effects occurring during an ultrasound procedure is indicated by a parameter known as the mechanical index, given by the equation [76]

(6) $$\mathrm{MI}=\frac{P}{\sqrt{f}}$$

where *P* is the peak negative pressure (PNP) and *f* is the ultrasound frequency. The PNP of medical ultrasound systems is generally insufficient to rupture homogeneous fluids [77]. However, in most biological fluids, certain discontinuities pre-exist that allow for the realization of cavitation at PNP values used for medical ultrasound. These discontinuities are called cavitation nuclei, and may exist as gases dissolved in the fluid or gas volumes stabilised at solid–liquid interfaces.

### 2.3. Instrumentation

The most common method of generating ultrasound waves is by using piezoelectric materials, which become electrically polarised when strained [78,79]. This generation of an electrical signal from the deformation of the material is called the direct piezoelectric effect. Conversely, by introducing these materials to an electric field, a deformation occurs by the inverse piezoelectric effect. The underlying aspect of the material responsible for these inverse phenomena is the asymmetric structure of the unit cells of the material [78,80,81]. When a piezoelectric material experiences a radiofrequency (RF) alternating electric field, it expands and contracts, creating regions of high and low pressure at its surfaces, which propagate as ultrasound waves. The earliest piezoelectric transducers for ultrasound generation utilised a single piezoelectric element [82], but, with the advent of array transducers (annular, linear, and matrix), dynamic focusing and beamforming with control electronics broadened the range of the capabilities of ultrasound [83].

**Figure 1:**
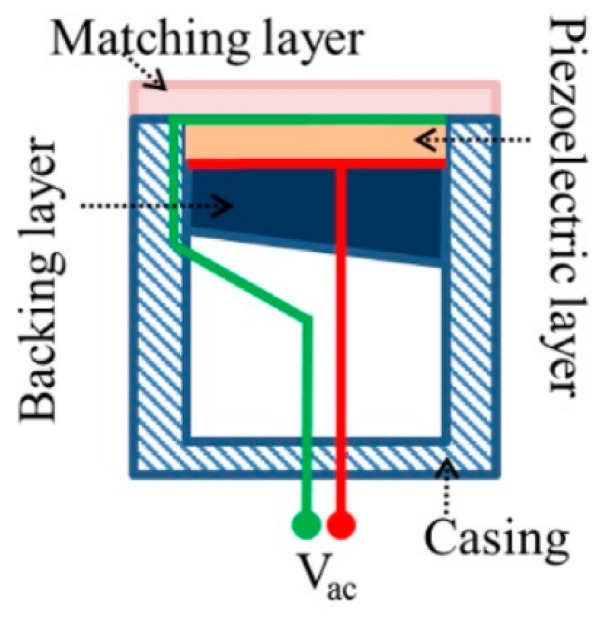
Schematic of transducer cross-section [86].

Several important components in ultrasound transducers may vary in size, shape, or material, depending on the intended applications [84,85]. The common construction though includes a layer of piezoelectric elements; electrodes; a matching layer to reduce the acoustic impedance difference between the piezoelectric elements and the interrogated material; a backing layer to mitigate wave interference caused by reflections for the rear surface of the elements; and an electrically insulating housing (Figure 1).

Generally, for a piezoelectric element operating in thickness mode, the element acts as a half-wavelength resonator [78]. The centre frequency of the transducer is thus related to the thickness of the transducer by [78]

(7) $$D=\frac{c_{p}}{2f}$$

with *D* being the element thickness, $c_{p}$ the speed of sound in the piezoelectric material, and *f* the centre frequency. Though a wide range of frequencies have been used for ocular ultrasound in the literature (Table 3), many have employed low frequency (≤100 kHz) ultrasound [87,88,89]. This has been done to emphasise mechanical bioeffects and simultaneously limit potentially damaging thermal effects [90].

## 3. Ultrasound-Mediated Drug Delivery

In addressing the low bioavailability and risk of side effects that systemic drug administration can pose, the encapsulation and binding of drugs within vesicles and to nano-/micro-particles provide benefits such as increased accumulation in diseased tissue structures and extended residence time at the site of action following administration [91]. This is in part due to the capacity of these carriers to reduce the drugs’ activity and interaction with other molecules and cells. As such, the ability for the encapsulated drugs to reach their intended destination is improved by evading clearance and immune responses, but, for effective release and activation, further stimuli may be necessary [92]. Additionally, even once systemically administered drugs have reached the vasculature of the targeted tissue via the bloodstream, the next requirement is that therapeutic dosages traverse tissue barriers and the membranes of the target cells. Ultrasound is increasingly being proven an efficacious method for overcoming both of these challenges through thermal and non-thermal effects. Though the majority of these studies are preclinical and have been carried out in animal models, their results appear promising for future translation to clinical trials.

**Figure 2:**
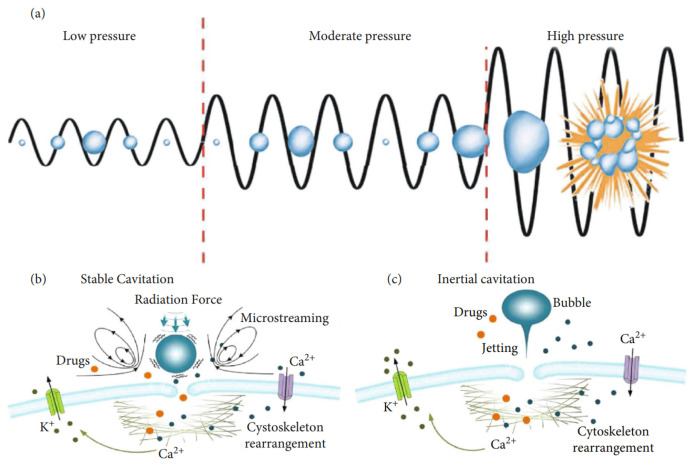
(**a**) Bubble action under varying acoustic pressure. (**b**) Non-inertial cavitation-generated microstreaming and shear stress on cell membrane. (**c**) Microjetting towards cell membrane under inertial cavitation [102].

While recent studies have demonstrated the effective use of acoustic radiation force for in vivo manipulation of objects and cells for theranostics [93,94], for targeted drug delivery, the largest body of existing research is devoted to the effects of ultrasound-induced acoustic cavitation. Although some attention has been given to the utilization of cavitation to release therapeutic agents from carriers [95,96], the phenomenon has thus far shown the greatest promise in transiently altering the structure of biological membranes and permitting intracellular delivery of drugs, genetic material, proteins, and other substances [97,98,99]. Still, the exact mechanisms responsible for the observed increase in permeability of tissues under the influence of acoustic fields are not entirely understood, but it has been mostly agreed in the literature that the cause is an amalgamation of primarily non-thermal effects that include the generation of fluid flows that produce shear stresses on cell membranes, expansion and contraction of gas bubbles attached to cells, causing the transient opening of pores, and fluid jets created by the implosion of gas bubbles puncturing cell membranes (Figure 2) [100,101].

In biofluids, it is not assured that cavitation nuclei density will be great enough to bring about meaningful therapeutic effects [103,104]. For this reason, it has become common practice to administer synthetic cavitation nuclei or nuclei-stabilizing particles in addition to the therapeutic agent [105]. Nuclei can be tailored with the capacity to facilitate stable or inertial cavitation, alter the cavitation thresholds of biological fluids, and even act as contrast agents for image-guided procedures.

**Figure 3:**
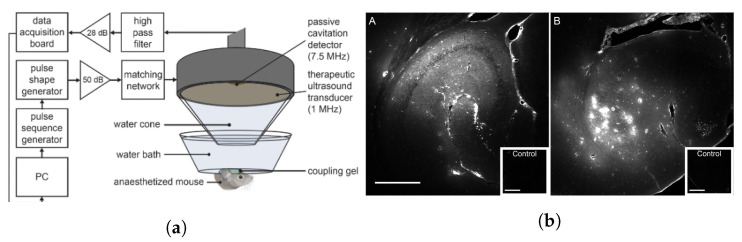
(**a**) Ultrasound and passive cavitation detector setup for mouse BBB disruption. (**b**) Fluorescent images showing dextran distribution in the hippocampus after (A) short pulse (pulse length = 5 cycles, PRF = 1.25 kHz) ultrasound treatment and after (B) long pulse (pulse length = 10,000 cycles, PRF = 0.5 Hz) ultrasound treatment [110].

An example of a physiological barrier that can be overcome by ultrasonic cavitation is the blood–brain barrier (BBB)—a semipermeable membrane consisting of epithelial cells, immune cells, pericytes, and astrocytes that collectively regulate the diffusion and transport of molecules between the bloodstream and brain/cerebrospinal fluid [106,107,108]. The selectivity of the BBB is crucial for the maintenance of homeostasis but severely inhibits CNS drug delivery. Over the last two decades, the obstruction posed by the BBB to effective treatment of neurodegenerative diseases and various brain tumours has led researchers to consider the potential benefits that ultrasound can offer to the existing interventions. Due to the sensitivity of this region of the body, there has been great emphasis on determining the safety of ultrasonic parameters for BBB disruption. As a result, many studies have adopted a low-intensity pulsed ultrasound protocol [109,110,111,112] since it has been found that there is a positive correlation between the duty cycle and neural tissue damage [113]. Figure 3 shows the experimental setup used by Morse et al. [110] in a study which demonstrated that low-energy, short-pulse focussed ultrasound combined with intravenous microbubble administration was effective at increasing neuronal uptake of labelled model drugs (Dextran, 3kDa) in rats by transiently disrupting the BBB with no observable tissue damage.

Similar in both structure and function to the BBB are the blood–retinal barrier (BRB) and the blood–aqueous barrier (BAB), which protect and regulate homeostasis in the eye. Both the BRB and BAB pose great resistance to the delivery of systemically administered therapeutic agents to ocular tissues, as will be further discussed in the following section.

## 4. Ocular Drug Delivery and Barriers

**Figure 4:**
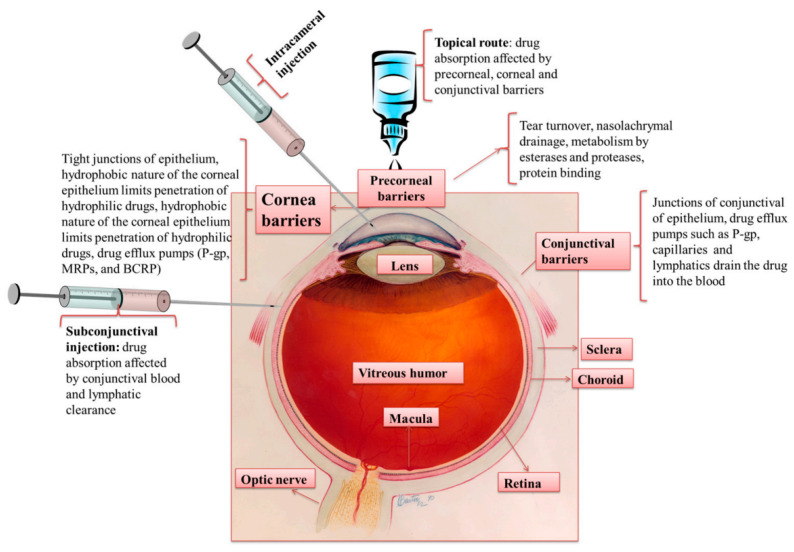
Routes of administration for ocular drug delivery and associated ocular barriers [114].

Within the literature, the eye is generally divided into two segments. The anterior segment comprises the cornea, conjunctiva, anterior sclera, ciliary body, lens, and aqueous humour, and the posterior segment includes the vitreous humour, posterior sclera, choroid, and retina. It is known that in both anterior and posterior segments, regardless of the route of administration, therapeutic agents encounter resistance in their transport to their intended site of action. The structures and the mechanisms responsible for this resistance are related to the anatomical and physiological barriers of the eye (Figure 4).

Currently, several methods of administering drugs for the treatment of ocular diseases exist and are employed based on the specific condition to be treated. However, each method has limitations (Table 2). Firstly, the systemic administration of drugs for treatment of ocular diseases through oral or intravenous routes is limited due to the difficulty in penetrating the blood–retinal (BRB) and blood–aqueous (BAB) barriers. The BRB is comprised of inner and outer BRBs. The inner BRB exists in the form of tight junctions between endothelial cells of the blood vessels supplying the retina, whilst the outer BRB consists of the retinal pigment epithelium—the outermost layer of the retina interfacing with the choroid [115,116].

Due to the large systemic dose, poor patient compliance, and the risk of side effects associated with systemic administration, topical administration has traditionally been the most common route for ocular drug delivery. Still, efficacious delivery of therapeutic agents to either segment of the eye via topical administration is hindered by numerous ocular barriers. The first encountered is the tear film. Produced in the lacrimal, conjunctival, and tarsal glands, as well as goblet cells, this fluid is secreted onto the surface of the cornea and conjunctiva and then dispersed by blinking and surface tension forces before collecting in the lacrimal sac and draining from the nasolacrimal duct. The reported rate at which this process occurs is approximately 1.2 μL/min [117], meaning that the film is entirely renewed every 5–6 min [118]. This rate is increased if the applied formulation causes irritation and induces increased lacrimal secretion production. With topical instillation, this process causes a majority of the dose to be washed from the eye surface into the nasal cavity, where it may be absorbed into the systemic circulation. The obstruction posed by the flow of the tear film is compounded by its chemical structure and properties. The outermost layer is comprised of lipids, which interface with the environment on one side (non-polar lipids), and an underlying aqueous layer on the other (polar lipids) [119]. A mucin layer is intermediate to the aqueous layer and the corneal epithelium [120]. This contrast of hydrophilic and lipophilic layers can impede the dissolution of drugs and therefore lower their bioavailability. This barrier has been proven to be surmountable to a degree with delivery systems such as lipid-based drug carriers in emulsion. The degradation of the water and oil phases of the emulsion can allow the transport of encapsulated therapeutics to the surface of the cornea [121].

**Table 2:** Common ocular diseases and conventional treatments.

Ocular Disease	Affected Tissue	Common Treatment	Limitations
Glaucoma	Optic nerve	Topical β-blocker [122]	Systemic side effects [123]
Macular degeneration	Retina	Anti-VEGF therapy [124]	Invasive [125]
Cataract	Lens	Phacoemulsiphication [126], Intravitreal anti-VEGF injections [127]	Invasive [128]
Diabetic retinopathy	Retina	Photocoagulation [129]	Invasive
Conjunctivitis	Conjunctiva	Topical antibiotics [130]	Drug clearance, bacterial resistance [131]

Beyond the tear film, the cornea itself also exhibits a layered structure with varying hydro- and lipophilicity. Furthermore, the tight junctions between the stratified squamous cells of the hydrophobic corneal epithelium may possess size-selectivity [132,133]. Another significant property of the cornea is its negative charge at physiological pH [134]. This is a property that has been frequently exploited in an attempt to increase the pre-corneal residence time of therapeutics [135,136].

**Figure 5:**
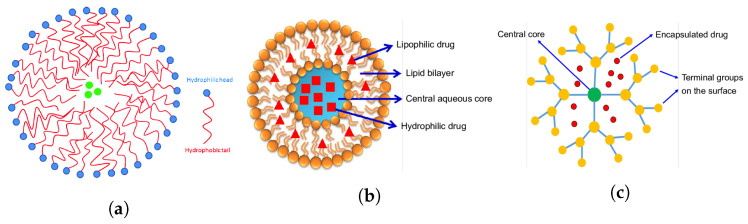
Structure of (**a**) drug-loaded (green) micelle [141], (**b**) liposome, and (**c**) dendrimer [142].

Nanotechnology for augmenting traditional therapeutic interventions targeting the anterior segment has been subject to extensive investigation. Nanoparticles, nanoemulsions, nano micelles, and dendrimers are some of the most common systems to have shown good efficacy for ophthalmology applications (Figure 5). Such systems can be tailored to possess specific physical, chemical, and electrical properties that positively impact the bioavailability of their cargo. For example, nanoparticle formulations instilled in the conjunctival sac have been found to increase drug concentration in the aqueous humour and prolong residence time when compared with free drug application [137]. Additionally, the amphiphilicity of nanomicelles has allowed the delivery of hydrophobic drugs in aqueous solutions, which is believed to reduce irritation and increase contact time with the cornea. A study has also shown that nanomicelles can be taken up by the corneal epithelial cells by endocytosis [138]. Dendrimers, on the other hand, are distinguished by repeatedly branching polymeric structures [139]. They have also been reported to increase the corneal permeation of drugs. In one study, the authors attributed this to the ability to increase drug solubility and thus create greater pre-corneal, free drug concentrations [140]. Such a concentration gradient can be important in diffusion across the cornea. It is also expected that positively charged dendrimers will interact with the negatively charged corneal surface to increase drug residence time.

Despite these novel drug-delivery systems, topical administration for drug delivery to the posterior segment is still regarded as largely ineffective [143,144]. This may be due to the clearance of drugs from the aqueous humour by aqueous outflow via the trabecular network or systemic absorption by the blood vessels of the iris and ciliary body [145]. Though there have been some studies indicating the potential for the accumulation of therapeutic concentrations of topically administered drugs, the most common method of posterior segment drug administration has been and continues to be intravitreal injection [145,146]. Intravitreal injections are an invasive procedure, but they can be safe when proper procedures are followed and patient-specific conditions are considered [147,148]. The risk of inflicting tissue damage and instigating or exacerbating ocular diseases is still present however, especially with repeated sessions, and thus much importance has been placed on increasing the efficacy of the procedure [149,150,151]. A method of doing so has been to use drug-delivery systems that reside in the intraocular tissues for extended periods and can allow the sustained release of therapeutic agents.

Much as the tear film is the initial site of administration for topically applied drugs, the vitreous humour is the first barrier that intravitreally administered drugs must overcome. The vitreous is a viscous, transparent fluid primarily consisting of water with collagen fibres and glycosaminoglycans in lesser concentrations [152]. It is also known to change with age, with young vitreous presenting a clear appearance and uniform viscosity. With age though, the collapse and thickening of collagen fibres, partial liquefaction of the vitreous, and reduction in overall volume can lead to issues such as retinal detachment and alterations of the pharmacokinetics of administered drugs [144].

**Figure 6:**
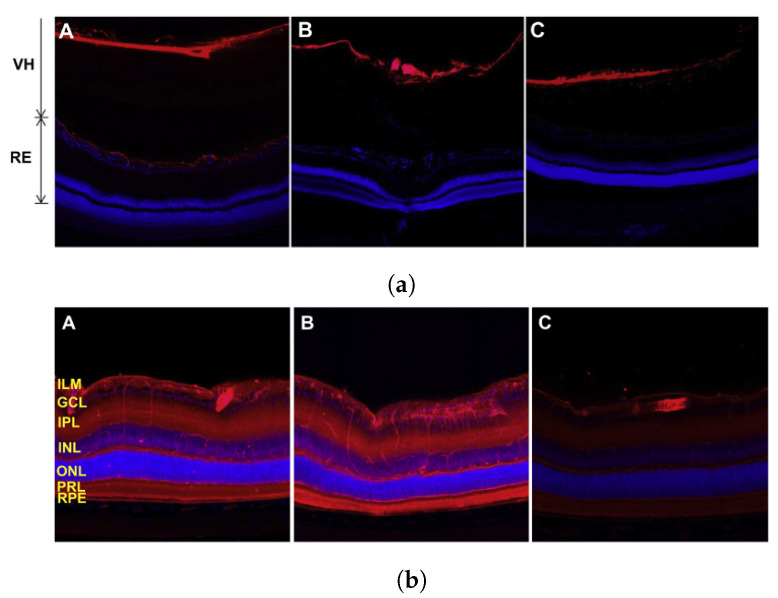
(**a**) Vitreal and retinal distribution of cationic polyethyleneimine nanoparticles 6 (**A**), 24 (**B**), and 72 (**C**) hours post injection. (**b**) Vitreal and retinal distribution of anionic hyaluronic acid nanoparticles 6 (**A**), 24 (**B**), and 72 (**C**) hours post injection [153].

As the vitreous humour has an overall negative charge, it has been shown in studies that the charge of intravitreally administered drugs will influence their distribution, with anionic substances being able to diffuse more easily than cationic ones [144]. Koo et al. [153] studied the movement of polymeric nanoparticles in the vitreous humour and reported that 6 h post-injection cationic polyethyleneimine nanoparticles were unable to diffuse from the site of injection, with similar observations 24 and 72 h post injection (see Figure 6a). Anionic hyaluronic acid nanoparticles, on the other hand, were effective in traversing the vitreous humour and reaching the retina, though were seen to have mostly cleared by 72 h (see Figure 6b).

In another study by Pitkänen et al. [154], investigating the delivery of genetic material to retinal pigment epithelial cells with non-viral vectors, they found that 20% and 6–15% of cationic polymer–DNA and lipid–DNA complexes, respectively, were taken up by cultured retinal pigment epithelial cells; whereas, in the presence of a layer of bovine vitreous, the uptake of both complexes was reduced to <2%. Hyaluronan solution also inhibited the transfection of the complexes, suggesting that this is one of the key components of the vitreous responsible for blocking cellular uptake.

Another barrier posed by the vitreous, though not fully understood, is the flow in the vitreous. Numerous studies have stated that the flow of vitreous humour is either negligible or non-existent [155,156,157,158]. However, more recently, through experiment and simulation, there has been emerging evidence that, in addition to the anterior outflow of the aqueous humour, there may be a posterior flow that is cleared through the retinal pigment epithelium. This effect may be more apparent with the liquefaction of the vitreous, and further investigation is required to determine the implications this would have for disease progression and drug delivery [152,158].

## 5. Ultrasound-Mediated Ocular Drug Delivery

There are various routes by which therapeutics can be administered and delivered to the eye. With topical administration, they may pass through the cornea or sclera to reach the anterior chamber. Alternatively, drugs in the bloodstream are required to pass through the BRB or BAB to become established in the anterior or posterior segment. Furthermore, intravitreally administered drugs must effectively diffuse through the viscous and negatively charged vitreous humour. For many of these routes, ultrasound, of various parameters (Table 3), has been shown to increase the transport of compounds across the respective barriers.

### 5.1. Ultrasound-Mediated Transcorneal Drug Delivery

**Figure 7:**
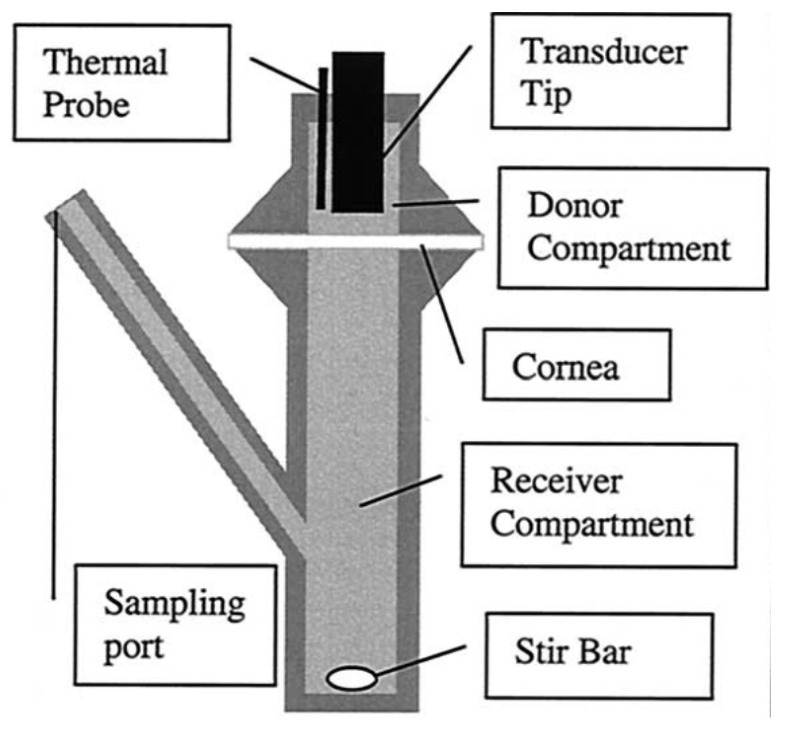
Diffusion cell setup used by Zderic et al. [70] to measure rabbit cornea permeability.

**Figure 8:**
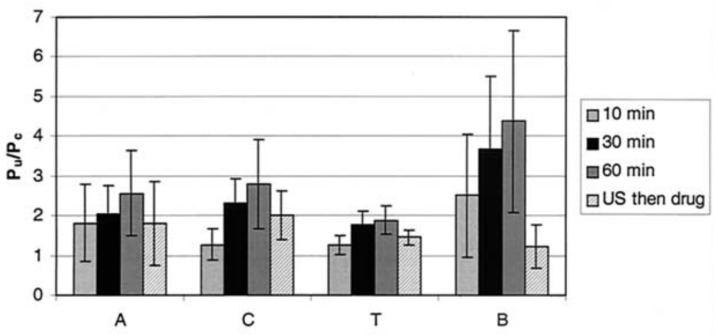
Ratio of permeability of treated cornea to control cornea to various drugs for various treatment durations in the presence of atenolol (A), carteolol (C), timolol (T), and betaxolol (B) [70].

**Figure 9:**
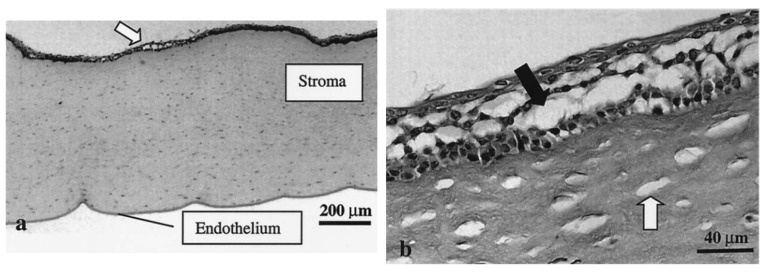
Microscope images of the cornea following a 60 min ultrasound treatment. The images show (**a**) partial detachment of the epithelium from the stroma and (**b**) the appearance of “bubble-like” structures in the epithelium (black arrow) and in the stroma (white arrow) [70].

An early study by Zderic et al. [70] was designed to determine the impact of 20 kHz, I_SATA_ = 2 W·cm^−2^ ultrasound (continuous mode) on the permeability of rabbit corneas to lipophilic beta-blocker medications. The experimental apparatus consisted of a diffusion cell (see Figure 7) with the excised cornea separating the donor and receiver compartments. The donor compartment was filled with the drug solution and ultrasound was applied for 60 min. Permeability of the cornea samples was determined using Fick’s law, and the ratio of the permeability of the sonicated cornea to the control cornea was presented as the permeability increase. The authors found that, with the application of ultrasound, the permeability of the rabbit corneas increased 2.6 times, 2.8 times, 1.9 times, and 4.4 times for atenolol, carteolol, timolol, and betaxolol solutions, respectively (Figure 8). With light microscopy, alterations in the cellular structure of the corneal epithelium and stroma were confirmed (Figure 9). The authors attested that this structural change was a primary cause of the increase in permeability and that it was possibly evoked by cavitation effects given the low frequency used. In the same study, the authors also sonicated the cornea sample for 60 min, then subsequently administered the drug formulation to the donor compartment once the ultrasound treatment had ended. This was carried out to find if the ultrasound had lasting effects on drug permeation. There was, however, reported to be a lesser increase in permeability measured in this case when compared with the 60 min combined ultrasound and drug exposure trials, with one drug showing no increase. From this, the authors inferred that, aside from the cellular disorganisation, other factors contributed to drug transport, such as convection from acoustic streaming and microstreaming. Despite the increase in drug diffusion, the authors stated that the protocol of this study was not optimal and that future studies should seek to determine the acoustic parameters most suitable for transcorneal drug diffusion and examine the reversibility of the observed structural changes.

Further investigation of the effect of varying acoustic parameters was undertaken in a later study by the same group using a similar experimental setup [159]. However, in this study, the curved shape of the cornea was maintained and hydrophilic drug solutions rather than lipophilic ones were used. An unfocused transducer was operated at frequencies of 400 kHz, 600 kHz, 800 kHz, and 1 MHz and intensities of 0.3, 0.5, 0.8, and 1 W·cm^−2^. Though a statistically significant increase in corneal permeation of dexamethasone (516.41 Da) with a trend of increasing permeation with a lower frequency (400 kHz) and greater intensity (1 W·cm^−2^) was seen, this was not observed with all drug solutions. This may in part be due to the hydrophilicity of the drug solutions. It is known that the stroma is a greater barrier to lipophilic compounds than the epithelium [160,161]. Similar to the authors’ previous studies, light microscopy was used to confirm that there was tissue damage in the epithelium but not in deeper tissues. The preservation of the structure of the hydrophilic stroma may have been the cause for a lack of significance in corneal permeability increase to lipophilic drugs.

With the recognition that the excision and preservation of animal corneas are likely to affect both structural and biochemical properties and perhaps influence the permeation of therapeutics in vitro, in vivo ultrasound-mediated transcorneal drug-delivery studies have also been conducted. Furthermore, these studies have also shed light on the regenerative capacity of corneal tissues following ultrasound-induced damage. With 5 min of exposure of 880 kHz ultrasound to rabbit corneas in vivo at intensities of 0.19 W·cm^−2^, 0.34 W·cm^−2^, and 0.56 W·cm^−2^, the aqueous humour concentration of topically applied sodium fluorescein was 2.4, 3.8, and 10.5 times greater, respectively, than the control with no ultrasound. A passive cavitation detector with a 5 MHz hydrophone was used to identify the incidence of cavitation when the transducer was operated at the given parameters. The authors found that stable cavitation occurred at all intensities, with inertial cavitation generated at and above 0.34 W·cm^−2^ [162].

The capacity of ultrasound to increase cornea permeability has also been studied for its potential use in the treatment of keratoconus—a disease that manifests in thinning of the cornea and resultant alterations in vision [163]. The treatment of this disease can call for intervention to increase crosslinking of collagen fibres in the corneal stroma to increase the stiffness of the tissue and prevent further degeneration. Surgery to achieve this involves the removal of the corneal epithelium and instillation of a riboflavin solution prior to ultraviolet radiation. This is invasive and thus patient compliance may be low, with risks of tissue damage or infection. In a study by Sun et al. [88], the impact of low-frequency, low-intensity ultrasound on the perfusion of a riboflavin solution into the corneal stroma was reported. Ex vivo and in vivo experiments were conducted in the study with porcine and rabbit eyes, respectively. In both cases, the eye was in contact with an adaptor containing the riboflavin solution and was integrated into a water bath to regulate the temperature during ultrasound treatments. The ultrasound transducer operating in continuous mode at 40 kHz was positioned 16 mm from the cornea, corresponding to the near field distance and transmitting through the riboflavin solution. The study reported the effect of exposure duration and mechanical index on the concentration of riboflavin in the cornea. A quantitative assessment of corneal absorption of riboflavin was obtained by scanning a Fluorotron fluorophotometer across the cornea, with corneal absorption defined as the area under the curve of the fluorescence intensity over the entire cornea.

The study showed that, of the three mechanical indexes used (0.2, 0.4, and 0.8), significant absorption of riboflavin into the corneal stroma was only achieved at MI = 0.8. In addition, compared with absorption in porcine eyes with the epithelium removed and no ultrasound treatment, this mechanical index provided similar absorption after a 30-min exposure. The ultrasound treatment also appeared to produce greater stromal penetration depth than passive diffusion in eyes with the epithelium removed. A comparison of treatment durations also showed a positive correlation between duration and riboflavin absorption, with the 10-min and 20-min exposures, resulting in 90% and 40% less absorption, respectively, than the 30-min exposure. Similar results were seen with in vivo experiments where a significant increase in riboflavin absorption was seen after 30 min of ultrasound with MI = 0.8, when compared with 15 min. The latter duration was still found to produce absorption comparable to longer durations of passive diffusion with the epithelium removed in the ex vivo eye. As was noted by the authors, riboflavin is normally exposed to the cornea for 30 min after epithelium removal. The incorporation of ultrasound into the procedure could shorten the duration of the treatment and achieve similar results by less invasive means, possibly reducing the risk of side effects and increasing patient compliance.

### 5.2. Ultrasound-Mediated Transscleral Drug Delivery

Although ultrasound has shown promise in increasing the transcorneal penetration of drugs to the anterior segment, further ocular barriers still impede diffusion to the posterior segment. As discussed above, intravitreal injections are commonly used to bypass these barriers, but, more recently, ultrasound has been explored as a needleless alternative for the delivery of drugs to the vitreous chamber via the sclera. The sclera is known to present several barriers, both static and dynamic, to the transport of molecules from the ocular surface to the vitreous and retina [164]. More recently though, low-frequency, low-intensity ultrasound has proved viable for reducing the effects of these barriers. In an in vivo study using New Zealand white rabbits, Suen et al. [165] found dextran (70 kDa) applied topically to the sclera did not reach the vitreous humour in any detectable concentration. However, in the presence of dextran, three applications of continuous wave ultrasound at a frequency of 40 kHz and intensity (SATA) of 0.12 W·cm^−2^ for 90 s each resulted in dextran concentrations in the vitreous of 0.022 μg/g, 0.28 μg/g, and 1.48 μg/g for successive applications. The ultrasound parameters used corresponded to a mechanical index of 0.20 and temperature measurements showed a negligible increase during the procedure. Seven days after the ultrasound treatment, the maximum concentration of topically applied dextran in the vitreous was significantly lower (0.024 μg/g) than during the ultrasound treatment session. After fourteen days, the topically applied dextran was not detected in the vitreous. This indicated that the mechanism responsible for increasing the penetration of dextran through the sclera was temporary and that the weakening of the scleral barrier was not a lasting effect.

In a later study by the same group, the authors attempted to identify and examine the underlying cause of the increased scleral permeability caused by ultrasound [87]. In this study, the authors hypothesised that cavitation was a requirement for ultrasound-induced sclera permeability increases. To test this hypothesis, ultrasound of varying mechanical indices was applied to the posterior sclera of a New Zealand rabbit in vitro. Using a spherical joint diffusion cell, the diffusion of bovine serum albumin protein across the tissue was measured, as the authors explain the protein has a similar molecular mass (65 kDa) to the clinically used anti-angiogenic agent ranibizumab (48 kDa). As with the previous in vivo study, a frequency of 40 kHz was used, with intensities between 0.002 and 1.8 W·cm^−2^. Passive acoustic cavitation detection was used to identify inertial and non-inertial cavitation at the tested intensities. The increase in the magnitude of broadband noise in the frequency spectrum at intensities of 0.38 and 1.8 W·cm^−2^ indicated the occurrence of inertial cavitation in this range. Peaks in the spectrum were seen at the fundamental frequency and half of the fundamental frequency, indicating the presence of non-inertial cavitation at all intensities. Fluorescence microscopy was used to measure the penetration of the bovine serum albumin protein into the sclera. It was found that lower intensities were more effective for increasing the penetration depth of the protein into the sclera. From this, the authors inferred that non-inertial cavitation was more effective at increasing scleral permeability than inertial cavitation, with an intensity of 0.05 W·cm^−2^ generating the greatest penetration depth and corresponding to a mechanical index of 0.133 at 40 kHz. This finding appears to reject the hypothesis of [166] that inertial cavitation plays a significant role in ultrasound-induced sclera permeability increases. In their study, Razavi et al. [166] used HIFU with a frequency of 1.1 MHz, a duty cycle of 2.5% and time-averaged powers of 0.5–5.4 W to sonicate the excised posterior sclera of rabbits in a diffusion cell. The authors found that a PRF of 100 Hz produced greater sclera permeability than 1 kHz. As the authors stated, this showed a positive correlation between inertial cavitation activity and sclera permeability. Although, it is possible that other factors, such as increases in acoustic streaming magnitude, were also responsible for the increase in sclera permeability [166].

Upon examination of the collagen network of the sclera with second harmonic generation imaging after ultrasound treatment, Suen et al. [87] found that there was no significant alteration of the collagen structure or arrangement, allowing the authors to conclude that such alterations are not a requirement for macromolecule penetration. Rather they stated that the chaotic flow caused by microstreaming at the boundaries of bubbles oscillating in the non-inertial cavitation regime was responsible for the increased penetration of bovine serum albumen protein. This appears to agree with the study by [166], which also confirmed that any damage observed in the sclera caused by ultrasound was indistinguishable from damage caused by sample processing.

There was also no significant heating of the tissue in the study by Suen et al. [87], with the temperature only increasing beyond 1 °C to 1.2 ± 0.8 °C at an intensity of 1.8 W·cm^−2^. Similarly, the duty cycle used by Razavi et al. [166] was selected to mitigate thermal effects. Temperature increases ranged from 0.5 to 4.5 °C for the lowest and highest intensities, respectively. The increased sclera permeability, despite only small temperature increases, is in agreement with the conclusions drawn by Lamy et al. [167]. In their study, Lamy et al. [167] investigated the concentration of sodium fluorescein (332 Da) in the vitreous humour following 60 min of topical exposure on the outer surface of the sclera of ex vivo rabbit eyes. Protocols included ultrasound treatment trials, in which continuous 880 kHz ultrasound was applied via the eye cup containing the sodium fluoroscein solution for 10 min; thermal treatment trials, where in lieu of the ultrasound source a heating probe was used to maintain a temperature similar to that produced by the ultrasound treatment for 10 min; and control trials where no ultrasound or thermal treatment was applied. The authors found that ultrasound treatment increased the concentration of sodium fluorescein in the vitreous by a mean factor of 1.44, compared with control trials. However, there was no significant difference in concentration between the control trials and thermal treatment trials. This suggested that the mechanism responsible for the increase in sclera permeability was primarily mechanical, with thermal effects being negligible.

### 5.3. Ultrasound-Mediated Blood–Retinal Barrier Disruption

**Figure 10:**
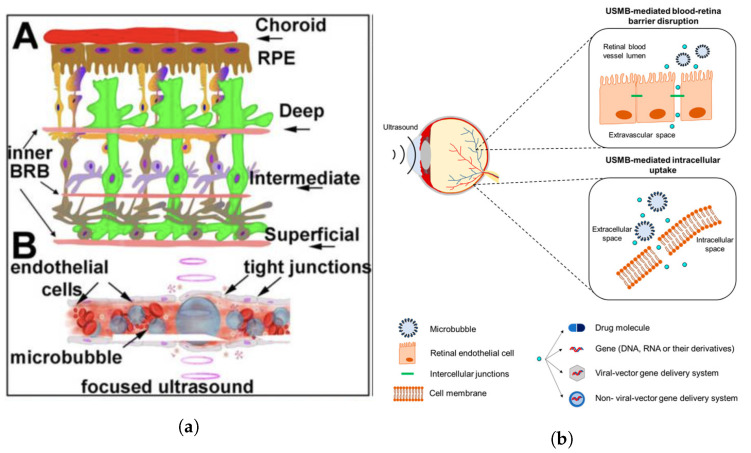
(**a**) (A) Structure of the vasculature supplying the retina, composing the inner BRB and the choroid adjacent to the retinal pigment epithelium (RPE) forming the outer BRB. (B) Illustration of the disruption of the BRB by gas bubble volume oscillation during ultrasonic cavitation. (**b**) The passage of substances through the resultant openings in blood vessels and cell membranes [169,170].

**Figure 11:**
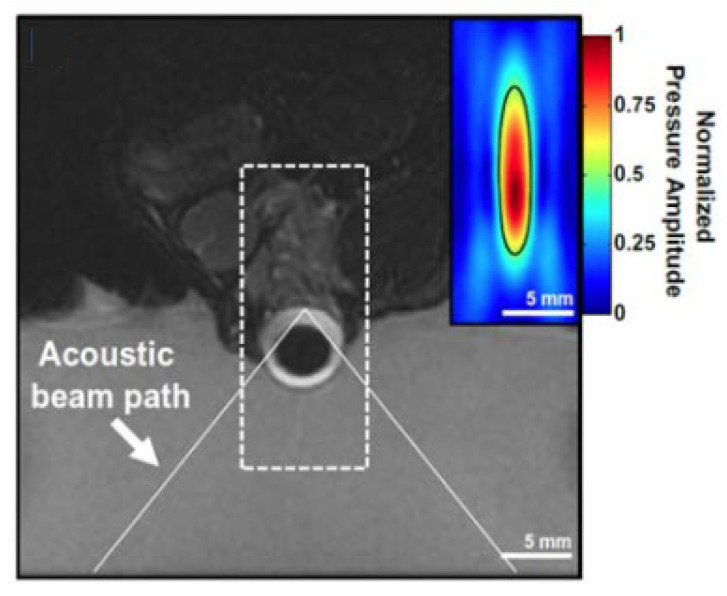
Acoustic pressure distribution in a rat eye using focused ultrasound for BRB disruption [168].

Analogous to how ultrasound has been used to disrupt the BBB transiently and allow improved delivery of therapeutics to the central nervous system, it has been shown that a similar effect may be achieved for the BRB (Figure 10). Though studies examining this phenomenon are still limited, Park et al. [168] showed that pulsed ultrasound focused to five overlapping volumes around the optic nerve of rat eyes in vivo, combined with intravenous microbubbles, resulted in greater uptake of an intravenously administered MRI contrast agent into the vitreous humour near the sonicated volume (Figure 11). A single element (d=100 mm), spherically curved (r=80 mm) transducer operating at 690 kHz was used in this study. The authors used peak acoustic pressure values of 0.81, 0.88, and 1.1 MPa to determine if there was a relationship between this parameter and BRB permeation. Indeed, it was reported that the intensity of the MRI contrast agent signal was greater after trials with increased acoustic pressure, though the results from the 0.81 and 0.88 MPa protocols were similar. However, 1.1 MPa exposure was found to produce tissue damage that was not detected with the lower two pressure values tested. The ultrasound beam was transmitted through the cornea and lens and therefore the authors accounted for the attenuation in the lens, stating that peak pressure at the retina was likely to be between 0.78 and 1.06 MPa. The exposure duration was 60 s at a PRF of 1Hz and pulse length of 10 ms.

Control trials, which still included the administration of the MRI contrast agent but where no ultrasound was applied, exhibited no increase in signal intensity in the retina or vitreous humour. Conversely, ultrasound treatment with all tested parameters resulted in signal enhancement in the retina and vitreous. The MRI contrast agent was readministered 3.5 h after the last ultrasound treatment and it was found that, while signs of increased BRB permeability were still observed by enhanced signal intensity, the magnitude of increase was significantly less than in the initial administration immediately after sonication. At 0.81 MPa, the average signal intensity enhancement in the region of interest was 30% immediately after ultrasound treatment. Administration of the contrast agent 3 h later resulted in an enhancement of <5%. This indicated the transient nature of the BRB disruption caused by ultrasound with these parameters.

It is noted that, in this study, the MRI contrast agent was only administered after sonication. It is therefore unclear if ultrasound treatment in the presence of the contrast agent would affect uptake into the vitreous. Furthermore, the authors highlighted the limitations of their study, such as the limited range of acoustic parameters tested and the potentially low sensitivity of the MRI technique used to minor BRB disruptions.

**Figure 12:**
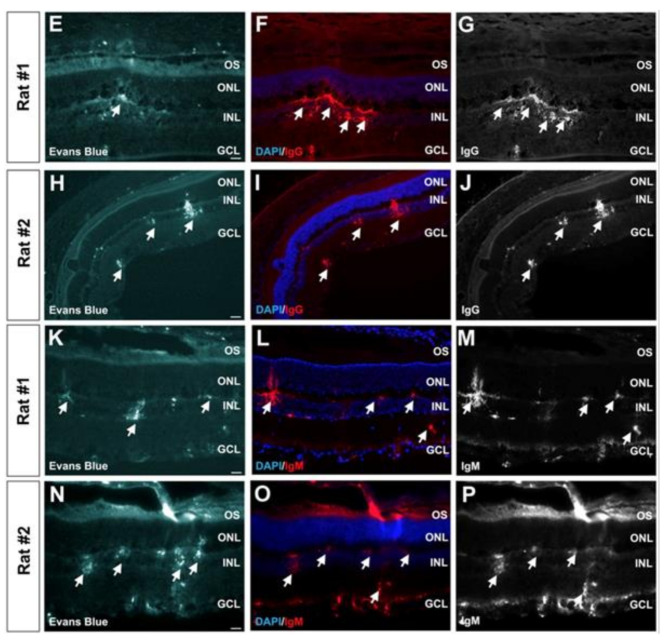
Photomicrographs showing points of accumulation (arrow heads) of Evans Blue dye (**left**), immunoglobulin G (**centre**), and immunoglobulin M (**right**) macromolecules in retinal parenchyma following ultrasound treatment [169].

In a study by Touahri et al. [169], the authors aimed to use focused ultrasound to deliver genetic material carried by adeno-associated viruses to the Müller glial cells of the retina, and to discern effective and safe parameters for this application. Ultrasonic parameters that were efficacious for BRB disruption in in vivo rats were initially established by examining the permeation of a systemically administered MRI contrast agent into the retina. A spherically curved (r = 60 mm) focused ultrasound transducer (d = 70 mm) operating at 1.1 MHz (PRF = 1 Hz, pulse length = 10 ms) was focused on the retina through the cornea. The pressure amplitude of the 10 ms bursts was increased until subharmonic emissions were detected by a hydrophone located at the centre of the ultrasound transducer, and then was reduced to 50% of this value for a total treatment duration of 120 s and a pressure range of 0.360–0.84 MPa. It was noted that this protocol, in combination with circulating microbubbles, had previously been shown effective in disrupting the BBB in rats [171]. MR images displayed increased contrast in the retina, indicating increased BRB permeability. Determination of the permeation of larger macromolecules through the BRB was conducted by systemic administration of Evans Blue dye, which binds to plasma albumin. After ultrasound treatment, the dye was administered via the tail vein. Thirty minutes later, the eyes were harvested, and microscopy showed that Evans Blue dye was present in the retina parenchyma proximal to the vessels innervating the retina in the inner nuclear layer and ganglion cell layer (Figure 12, left). This suggested the transport of macromolecules across the inner BRB. In contrast, the authors noted that there was no evidence of permeation across the choroid and outer BRB. The increased permeability of the inner BRB to larger macromolecules was confirmed by increased concentrations of immunoglobulin G and immunoglobulin M in the retinal parenchyma (Figure 12, centre and right respectively)—antibodies with reported molecular masses of 150 kDa and 970 kDa, respectively. Minimal damage was observed in the retina with the focused ultrasound protocol described.

The same procedure was followed for the investigation of the effect of focused ultrasound on the transduction of Müller glial cells by a viral vector that does not readily cross the blood–retinal barrier under normal conditions. Adeno-associated viruses carrying a glial fibrillary acidic protein promotor and a mCherry reporter were systemically administered after ultrasound treatment in combination with microbubbles. Three weeks later, the eyes were harvested and examined. It was found that, in the eyes of rats that received doses with virus concentrations of $2.5\times 10^{9}$ genome copies per gram, astrocytes and Müller glial cells expressed the mCherry protein, indicating transduction. The increased mCherry expression was not detected in rat eyes when a lower virus concentration was administered ($1.25\times 10^{8}$ genome copies per gram) or when ultrasound was not applied.

Leakage of macromolecules from the bloodstream through the BRB after ultrasound treatment was also looked at by the administration of Evans Blue dye that binds to albumin. The detection of this dye in the retina would indicate the leakage of plasma proteins through the BRB. Evans Blue dye was found to be present in the inner nuclear layer and ganglion cell layer of the retina. In contrast, the authors stated that there was no evidence that the dye entered the retinal parenchyma through the choroid. Thus, the results suggested that ultrasound treatment in combination with intravenous microbubbles increased the permeability of the inner BRB to plasma albumin protein, but the same effect was not realised for the outer BRB. Regarding the safety of the procedure, immunofluorescence microscopy showed an increase in intensity at the site of dye permeabilisation in only one of the six rats tested.

**Figure 13:**
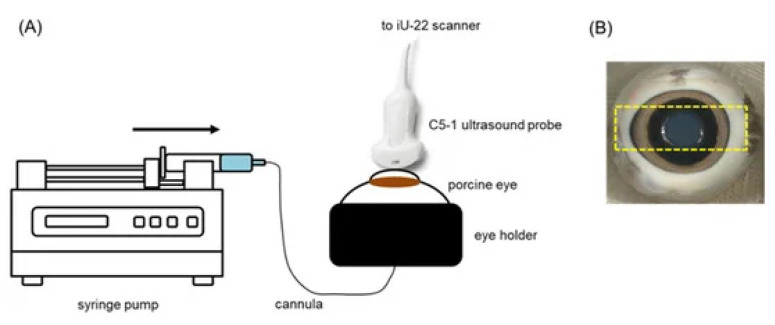
Diagram of (**A**) the experimental setup and (**B**) probe placement for ultrasound treatment with microbubble perfusion in an ex vivo porcine eye to investigate drug delivery to the retina [172].

Ultrasound treatment in combination with circulating microbubbles has also been found to compromise the BRB and increase intracellular delivery of small (600 Da dye) and large (4 and 20 kDa dextrans) molecules to the adjacent cells in larger ex vivo porcine eye models. Rousou et al. [172] used a clinical pulsed-wave Doppler ultrasound system with the transducer coupled to the cornea (Figure 13) to sonicate the retina of ex vivo porcine eyes perfused with a solution of microbubbles, a fluorescent green stain, and fluorescent dextrans via the ophthalmic artery. Control trials were identical, other than the omission of microbubbles from the solution.

The ultrasound treatment was carried out for 2 min at a frequency of 2.5 MHz and mechanical indexes of 0.4 and 0.8 (0.3 and 0.6 MPa, respectively). Perfusion with a solution containing the fluorescent stain and dextrans was continued for 30 min after the ultrasound treatment prior to histological analysis.

**Figure 14:**
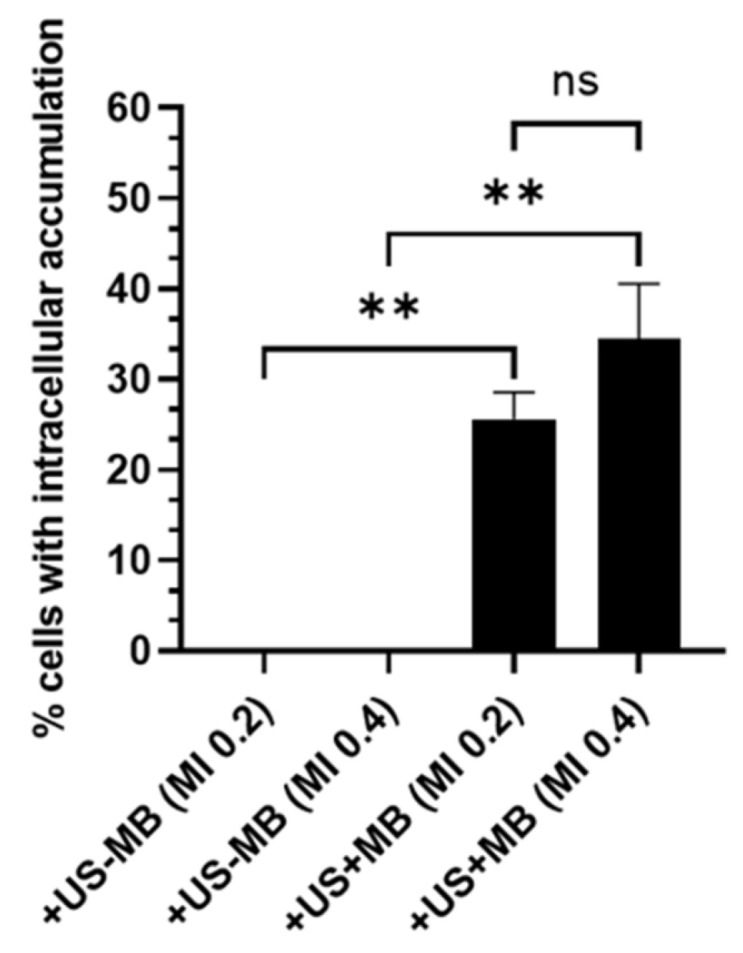
Percentage of cells lining examined blood vessel exhibiting accumulation of dye and dextrans [172]. ** = *p* < 0.005; ns = not significant.

A comparison of contrast ultrasound images showed that there was a greater reduction in contrast after the ultrasound treatment in the 0.4 mechanical index trials. This was deemed to suggest the occurrence of inertial cavitation at this pressure. With fluorescence microscopy, the intracellular uptake of the dye and dextrans was confirmed in cells lining the blood vessels in the retina and choroid, with dextrans present in the cytosol and nucleus. However, there was no evidence of permeation into the retina or choroid parenchyma. It was also shown that there was no detected uptake and accumulation of the dye or dextrans when microbubbles were absent from the solution (see Figure 14).

Though this study shows that intracellular delivery of macromolecules can be achieved with minimal and reversible tissue alterations, through ultrasound treatment in combination with intravenous microbubbles, the employed protocol is likely to be insufficient for delivery to deeper ocular tissues, as penetration of the administered dye and dextrans of both sizes to cells is outwith the blood vessels of the retina and choroid.

**Table 3:** Ultrasound parameters used in UMODD studies.

Study	Tissue	Effect	Frequency	Intensity (or Power)	Duty Cycle	MI	Duration	Drug/Model
[172]	Retina	Increased intracellular model drug accumulation.	2.25 MHz	-	-	0.2, 0.4	2 min	SYTOX green, TRITC dextrans
[173]	Sclera	Increased permeability at highest frequency.	400 kHz, 3 MHz	1 W·cm^−2^	100%	-	5 min	Avastin
[166]	Sclera	Increased permeability.	1.1 MHz	0.5–5.4 W	2.50%	-	10 min	Sodium fluorescein
[174]	Sclera	Increased drug penetration.	1 MHz	0.5 W·cm^−2^	100%	0.14	5 min	FITC-BSA-SFNP
[70]	Cornea	Increased permeability.	20 kHz	2 W·cm^−2^	14.3%	-	10, 30, 60 min	Atenolol, carteolol, timolol, betaxolol
[168]	Retina	Increased permeability. Retinal damage at 1.1 MPa.	690 kHz	-	1%	0.96, 1.06, 1.32	1 min	Magnevist
[159]	Cornea	Increased permeability.	400 kHz–1 MHz	0.3–1.0 W·cm^−2^	100%	-	5 min	Tobramycine, dexamethasone, sodium fluorescein

### 5.4. Ultrasound-Mediated Intravitreal Diffusion

As mentioned above, intravitreal injections are a common method of bypassing the ocular barriers that inhibit the penetration of topically applied therapeutics to the posterior segment. Despite this, vitreous humour is rarely the target for injected drugs. It is thus still necessary for the drugs to diffuse to the target tissue—often the retina. This is hindered by the barriers posed to molecules based on size and charge, as discussed above. Since injecting therapeutics at proximity to the site of pathology may require deep insertion of the needle near fragile tissues, it would be more desirable to administer the therapeutics at a shallow location and allow them to diffuse to the intended site of action to mitigate physical damage caused by the needle. Though limited, there are now studies that have evaluated the application of ultrasound to increase the diffusion of intravitreally administered substances through the vitreous humour [175,176].

Huang et al. [175] examined the effects of intravitreal and transscleral ultrasound on the penetration of hyaluronic-acid-modified human serum albumin nanoparticles through the neural retina to the retinal pigment epithelium and on the diffusion of the nanoparticles in the vitreous of ex vivo bovine and porcine eye models. After exposure with 1MHz continuous wave ultrasound with an intensity of 0.5 W·cm^−2^ for 30 s, retinal penetration and diffusion in the vitreous were both found to increase. The intensity was chosen to elicit ultrasonic cavitation and its associated bioeffects. Posterior eye cups were produced by removing the anterior ocular tissues and dividing the eye along the equator. For retinal penetration experiments, the vitreous was also removed. The nanoparticle suspension was added to the eye cups, and ultrasound was applied either coupled to the exterior of the sclera (transscleral) or directly into the vitreous chamber via the suspension (intravitreally). With intravitreal ultrasound, it was found that fluorescence intensity was significantly greater than for passive diffusion alone (no ultrasound). The authors also applied ultrasound and nanoparticles separately to identify any lasting effect of the ultrasound protocol on nanoparticle penetration. It was found that, when the nanoparticle suspension was instilled after ultrasound treatment, there was no significant difference in retinal penetration compared with the no-ultrasound trials. This suggested that streaming effects or acoustic radiation forces were influencing nanoparticle penetration through the neural retina. By repeating ultrasound exposures with 15-minute intervals, it was found that, compared with a single ultrasound exposure, neural retina and RPE/choroid fluorescence intensities increased 1.20- and 1.54-fold after two exposures, and 1.34 and 1.62 fold for three exposures, respectively, implying a nonlinear increase in penetration with repeated exposures.

Similarly, transscleral ultrasound also resulted in increasing neural retina and RPE/ choroid fluorescence intensities with repeated exposures, though to a lesser degree than that with intravitreal ultrasound. The authors speculated that this may have been due to energy absorption in the sclera and uvea with transscleral applications. The direction of ultrasound propagation being radially inward towards the centre of the globe rather than towards the retina, as in the intravitreal experiments, may also have been a factor contributing to the discrepancy [175].

For nanoparticle diffusion in the vitreous humour, only the transscleral ultrasound protocol was used. This was in part due to the authors deeming this method more clinically relevant. A total of 20 μg of the nanoparticle suspension was injected into the vitreous of intact ex vivo bovine eyes. Fluorescence microscopy found that, in the absence of ultrasound, the bolus did not disperse from the site of injection effectively. In contrast, after three 30-second exposures of ultrasound of the same parameters used for the retina permeation experiments, the fluorescence intensity at the site of injection was significantly reduced, evidencing that ultrasound increased the diffusion of the intravitreally injected nanoparticles.

Huang et al. [175] also quantitatively analysed ultrasound-mediated nanoparticle diffusion in the vitreous of ex vivo porcine eyes. Ultrasound was applied on the superior surface of the globe in line with the equator. After ultrasound treatments, the eye was divided in half through the cornea orthogonal to the equator. Results showed that the distribution of the fluorescence was more uniform in the ultrasound-treated eyes, whereas it appeared that the injected bolus did not effectively diffuse to the opposite half of the eye by passive diffusion.

The maximum temperature rise in the ocular tissues during the experiments was reported to be 1.3 °C. The temperature change was reported to be localised to the site of ultrasound application, with the temperatures of the surrounding tissues remaining constant, suggesting that thermal effects were not responsible for the observed changes in nanoparticle mobility. Moreover, with light microscopy, it was confirmed that there was no significant damage to the ocular tissues as a result of the ultrasound treatment.

As was acknowledged by the authors, it is unclear if, or to what degree, convective flow, which occurs in vivo, would affect the mobility of intravitreally administered nanoparticles both with and without ultrasound treatment.

In a later study by Thakur et al. [176], it was proposed that ultrasound could be a viable technology for increasing the magnitude and directionality of the diffusion of intravitreally administered nanobubbles. Again, ex vivo bovine and porcine eyes were obtained for this study. The ultrasound parameters were set at a frequency of 1 MHz, an intensity of 2.5 W·cm^−2^, an exposure duration of 60 s, and 50% and 100% duty cycles for the porcine and bovine eyes, respectively. The ultrasound source was positioned on either the sclera, at the pars plana, or the cornea. In both eye models, 100 μL of the nanobubble formulation was injected into the vitreous, posterior to the lens via the pars plana. In the bovine eyes and control eyes, optical fluorescence spectrometry was used to image the distribution of the injected nanobubbles following ultrasound treatment. The porcine eyes, on the other hand, were snap-frozen in liquid nitrogen in a procedure similar to that followed by Huang et al. [175], though in this study the eye was divided into four quadrants, consisting of the anterior quadrant nearest to the transducer; the anterior quadrant farthest from the transducer; the posterior segment nearest to the transducer; and the posterior quadrant farthest from the transducer. With the bovine eyes, only distribution in the anterior or posterior halves of the vitreous were considered and so only corneally applied ultrasound was examined. Both cornea and sclera applications were evaluated in the porcine eyes to investigate the impact of transducer position on anterior/posterior and lateral diffusion.

With regards to the bovine eyes, the authors reported that, with increasing ultrasound treatment cycles, the percentage of the injected nanobubble formulation in the posterior half of the vitreous increased significantly to 28.5 ± 8.5%, 45.1 ± 11.4%, and 47.8 ± 15.2% after one, two, and three cycles, respectively. In contrast, there was no observed diffusion of the injected bolus into the posterior half in the absence of ultrasound.

In the porcine eye models, the results showed that nanobubble diffusion was dependent on the transducer position. Scleral ultrasound resulted in greater lateral movement from the temporal anterior quadrant to the nasal anterior quadrant (56.5 ± 8.9%). Corneal ultrasound, however, produced greater transport of the nanobubbles from the temporal anterior quadrant to the temporal posterior quadrant (36.3 ± 4.2%).

Within their discussion, the authors attributed this increase in directional diffusion to the generation of ultrasonic streaming. It was also stated that streaming was not observed when using nongaseous liposomes and dyes. This appears to disagree with the findings of Huang et al. [175], who observed ultrasound-induced migration that was attributed to streaming, though this may be a result of the difference in intensities employed in the two studies.

## 6. Conclusions and Outlook

Though the use of ultrasound for increased ocular drug delivery efficacy has been shown to hold promise within the existing literature, it appears to still be in a preliminary stage of development and, as such, there are still challenges and limitations that future studies may seek to rectify. For example, parameters such as operating frequency, peak pressure, duty cycle, exposure time, and administration of contrast agents have been shown to vary across studies (Table 3). A better understanding of how such variation in these parameters impacts the safety and effectiveness of the technique is required before it can be introduced into the clinical setting. However, as suggested by [177], it may be more appropriate to focus on the further development and refinement of means of monitoring the mechanical and thermal effects occurring within the region of interest, as these are ultimately responsible for the increase in drug delivery effectiveness but also potentially for undesirable tissue damage. In addition, it is possible that acoustic field measurements that are taken in a homogeneous, isotropic medium such as a water tank, as was reported in some studies, may not give a true representation of the acoustic field established in eye models, either in vitro or in vivo [172]. Particularly for in vivo studies, the acoustic field could be sensitive to transducer placement, which may affect attenuation, refraction, and reflection from structures such as the lens or orbital bones and create complex interference patterns [168].

Thus far, a majority of studies on ultrasound-mediated ocular drug delivery have used rodent models. Instead, it may be prudent for future work to focus efforts on demonstrating ultrasound-mediated drug delivery in eye models more closely related to human eyes, such as porcine eyes. Inter- and intraspecies variability between ocular tissue samples obtained for the reviewed studies could also be a cause of discrepancies in results [70]. As with ultrasound systems for other medical procedures, safety and quality assurance will require a standard for device calibration. Future work may look at the possibility of developing ocular-tissue-mimicking phantoms that replicate the properties (e.g., acoustic and thermal) of certain ocular tissues for the testing, comparison, and calibration of ultrasound systems for ocular drug delivery. Al-Sadiq et al. [178], Thakur et al. [179] have reported methods of producing gel phantoms to mimic the sclera and vitreous humour of the eye. Further characterisation of properties such as thermal conductivity and specific heat capacity may allow for useful and consistent evaluation of the impact of variables such as coupling agent and ultrasound parameters on temperature changes during ultrasound treatment for drug delivery. A possible intermediate step may be the combination of ex vivo ocular tissues and tissue-mimicking materials, similar to that used by [180].

Regarding recent studies examining the use of ultrasound for the manipulation of intravitreally injected substances, it is noteworthy that, so far, the studies, such as those by Huang et al. [175], Thakur et al. [176], appear to have only examined the potential of using acoustic forces to move the substances from regions of high concentrations at the site of injection to low concentrations diffused throughout the vitreous. Future research may seek to determine if the inverse action of concentrating substances that are already diffused within the eye to predetermined regions can be achieved. This would likely require the establishment of standing wave patterns created by reflections from anatomical features or from multiple transducer systems.

Finally, as some studies have shown that the greatest increase in drug delivery occurs when the drug formulation is applied during ultrasound treatment rather than post-treatment, suggesting that transient mechanical effects are mostly responsible, repeated or prolonged treatments may still be required for meaningful therapeutic concentration increases, which could be a challenge in clinical settings [177]. In the future, a combination of the ultrasound procedures discussed above could be effective for overcoming several ocular barriers in a single treatment. Such a method could facilitate the diffusion of therapeutics through superficial ocular tissues and thereafter increase diffusion and directionality in the main chambers of the eye and maximise the effectiveness of the treatment.

## Abbreviations

The following abbreviations are used in this manuscript:

UMODD	Ultrasound-mediated ocular drug delivery
BBB	Blood–brain barrier
BRB	Blood–retinal barrier
BAB	Blood–aqueous barrier
TRITC	Tetramethylrhodamine
FITC-BSA-SFNP	Fluorescein isothiocynate-labelled bovine serum albumin-loaded silk fibroin nanoparticles
